# Promoting adherence to medicines: possible lessons for Canada?

**DOI:** 10.1186/s13584-017-0188-6

**Published:** 2017-11-20

**Authors:** Nigel S. B. Rawson

**Affiliations:** 1 Eastlake Research Group, Oakville, ON Canada; 2 Canadian Health Policy Institute, Toronto, ON Canada; 3 Fraser Institute, Vancouver, BC Canada

**Keywords:** Adherence, Medications, DTCA, Canada

## Abstract

Non-adherence to medication regimens is a major issue that can negatively impact patient health and wastes health care system resources. This commentary considers whether approaches to strategies undertaken in Israel to promote adherence could be viable in Canada. The structure of the Canadian health care system and budgetary constraints make new initiatives similar to those in Israel seem unlikely in Canada without some compelling stimulus.

## Commentary

Non-adherence to medication regimens is a major problem that can negatively impact patient health and wastes health care system resources. A lack of adherence has been shown to result from multiple factors related to different aspects of a health issue that include disease characteristics (adherence is usually higher when a serious ongoing disease is being treated), available treatments, a lack of therapeutic effect, the occurrence of an adverse reaction, health care system effects, and patient-related and physician-related factors [1] as well as economic barriers [2].

Regulatory strategies undertaken in Israel in recent years to encourage drug adherence are reported by Schwartzberg et al. [3]. These include revising old regulations to improve advertisements for over-the-counter medications, and the introduction of new regulations allowing disease awareness campaigns that provide information about the availability of treatments but not the promotion of particular products via direct-to-consumer advertising (DTCA). In addition, medical information centres approved by the Ministry of Health and funded by pharmaceutical companies have been established to offer information on the disease, medication and correct way to take the medication and to perform home visits to supply training on appropriate medication use. It would have been interesting if evidence had been included about the impact of the strategies.

Numerous arguments exist for and against DTCA of specific products [4], but most industrialized countries, including Israel, do not permit such advertising because it can and has been abused. Schwartzberg et al. [3] believe that DTCA leads to “disease mongering” and present some examples that they consider exemplify this practice, which come from a series of essays by anti-pharmaceutical industry academics.

Israel has advantages in its ability to promote adherence to medications. First, it is a small country with a modest population of less than nine million and a centralized government. Second, universal health coverage is provided to all citizens and permanent residents through four competing, non-profit health maintenance organizations that cover all services including prescription medications. Third, the Pharmaceutical Division of the Ministry of Health is not only the country’s regulatory authority but also the governing authority of the pharmacy profession.

In contrast, Canada, the second largest country in the world in terms of its land area with a population of 36 million, is a federation of 10 provinces and three territories of widely differing sizes, populations and legislative philosophies. The provision of health care services is a provincial and territorial government responsibility overseen by the federal government, which is the country’s regulatory authority for the approval and safety of medications. Separate provincial and territorial pharmacy regulatory bodies regulate the practice of pharmacy and operation of pharmacies in their respective jurisdictions.

Unlike every other country in the world with a universal government health insurance system for physicians, hospitalizations and laboratory services, Canadian federal, provincial and territorial governments do not cover all prescription drugs for patients in the community. Reimbursement for drugs is available through government-funded plans and private insurance paid for by individuals or cost-shared with employers, unions or associations.

Government drug plans, which offer a degree of insurance to about a third of Canadians, are mainly designed to provide coverage for seniors, social assistance recipients and some special groups, such as cancer patients, or when costs are deemed to be catastrophic. The government plans have a labyrinthine system of deductibles, copayments and premiums and, for many drugs, special or restricted access criteria or therapeutic substitution that result in variation in patient eligibility, out-of-pocket expenses and coverage [5]. These factors have led to significant inequalities in medication access and coverage across Canada.

Non-adherence has been demonstrated in Canada in the treatment of many chronic diseases, including those of the cardiovascular, respiratory, central nervous, gastrointestinal, skeletal and ocular systems. In some studies, a high proportion of patients have discontinued treatment after only one prescription. Most of these studies have been performed in a single province. Similarly, efforts to improve adherence have been provincially-focused, with most being short-term academic studies. This approach is typical of Canada when it comes to improving health care practices. Indeed, Bégin, a respected academic and a former federal Minister of Health, and her colleagues have called Canada “a country of perpetual pilot projects” [6] where proven projects are seldom moved into stable, funded programs and the outcomes of pilot projects are rarely transferred across jurisdictions.

Canada does not permit DTCA of prescription medications, except for vaccines. Nevertheless, in 2000, a change in the interpretation of the policy governing advertising occurred without public or parliamentary debate that allows direct-to-consumer information advertisements, which state the drug’s brand name without making health claims or inform consumers of new but unspecified treatment options [7]. These advertisements include ones that urge patients to ask for brand name medications rather than the generic versions to which public and private insurers often limit their coverage in order to contain costs; if patients ask for the brand name product, they may pay more for their prescription.

Since DTCA is legal in the United States and many Canadians watch American television channels and use American internet sites, Canadians are, in reality, exposed to a high level of prescription brand name drug advertising. As a result, Canadians frequently become aware of new drugs that are available in the United States but, due to generally later regulatory submissions in Canada and longer times taken to review and approve new drugs by Health Canada [8], are commonly available later (sometimes much later) in Canada. This often raises concerns among Canadian patients seeking medications for conditions for which current therapy does not exist or has limited effectiveness.

## Conclusions

The Government of Canada could introduce regulations that would require more strictly controlled advertisements, although Canadians would continue to be exposed to American influences. Canadian pharmacy and medical schools have drug information centres, but they are frequently underfunded and their services are generally limited to health care professionals. Provincial and territorial governments could establish larger and better resourced third party medical information centres, like those in Israel, to provide disease, medication and appropriate use information and to perform home visits to supply training on medication use.

However, the legislative structure of Canada and its health care system and the lack of a national plan that provides insurance coverage for all drugs to all residents, together with budgetary constraints, make new initiatives of this kind seem unlikely. Nevertheless, the ever-increasing costs of medications and the need to maximize the benefits of medications paid for by public and private insurers may eventually provide enough motivation for a coordinated public-private insurance program to promote medication adherence.

## Abbreviations

DTCA: Direct-to-consumer advertising
